# Different patient satisfaction levels between the first and second knee in the early stage after simultaneous bilateral total knee arthroplasty (TKA): a comparison between subjective and objective outcome assessments

**DOI:** 10.1186/s13018-017-0605-0

**Published:** 2017-07-26

**Authors:** Shuai Huang, Xing Li, Yubo Tang, Sunny Stiphan, Bin Yan, Peiheng He, Dongliang Xu

**Affiliations:** 1 Department of Joint Surgery, The First Affiliated Hospital of Sun Yat-sen University, 58# Zhongshan 2rd Road, Guangzhou, 510080 Guangdong Province China; 2 Department of Pharmacy, The First Affiliated Hospital of Sun Yat-sen University, Guangzhou, 510080 China; 3 Department of Orthopaedic Surgery, The First Affiliated Hospital of Sun Yat-sen University, 58# Zhongshan 2rd Road, Guangzhou, 510080 Guangdong Province China

**Keywords:** Simultaneous bilateral TKA, The first and second knee, Satisfaction

## Abstract

**Background:**

Simultaneous bilateral total knee arthroplasty (TKA) is an effective treatment option and safe for properly selected patients. The purpose of this study was to evaluate whether patients have different satisfaction levels between the first and second knee in the early stage after simultaneous bilateral TKA.

**Methods:**

We retrospectively reviewed 46 patients who underwent simultaneous bilateral TKA by a single surgeon in our department between March 2013 and March 2015. The surgeon typically performed first-side TKA (right knee), followed by TKA on the left knee. Tranexamic acid (TXA) (10 mg/kg) was given intravenously, and the tourniquet was released after wound closure. The preoperative KSS, ROM, and pain visual analog scale (VAS); the objective parameters including drainage volume and swelling evaluated by the circumference of the 10 cm above the patella; and the preoperative and postoperative (1st, 3rd, and 7th days) subjective parameters including pain, satisfaction VAS, and patient satisfaction of the first and second surgeries for each knee were analyzed.

**Results:**

In simultaneous bilateral TKA, compared with the second-side TKA (left knee), the first-side TKA (right knee) had a lower mean drainage volume (*p* < 0.05), but the swelling of the knee was higher on the 1st, 3rd, and 7th postoperative days (*p <* 0.05). Moreover, the first-side TKA was scored lower in satisfaction VAS but higher in pain VAS at the 1st, 3rd, and 7th postoperative days. The patient satisfaction scores indicated 2 (4.4%) of the 46 patients scored first-side TKA higher than second-side TKA, 34 (73.9%) of the 46 patients scored second-side TKA higher than first-side TKA, and 10 (21.7%) of the 46 patients scored their satisfaction as the same for both knees.

**Conclusions:**

This research study found that there was better patient satisfaction with the second knee in the early stage after simultaneous bilateral TKA, which may provide some considerations for surgeons choosing simultaneous bilateral total knee arthroplasty for patients with osteoarthritis in both knees.

## Background

Osteoarthritis of the knees could seriously affect the health and quality of life for elderly persons across the world. When the disease develops later in life, total knee arthroplasty (TKA) is used to reduce pain and improve quality of life and function in patients [1, 2]. However, a lot of patients who have bilateral disease need bilateral TKA to achieve their optimal functional results [3]. Staged-bilateral TKA and simultaneous bilateral TKA are used on the basis of the patient’s physical condition and the surgeon’s experience. Compared with the staged-bilateral TKA, several research studies have reported that simultaneous bilateral TKA increased complication rates including gastrointestinal complications, deep-vein thrombosis, pulmonary embolism, fat embolism, cardiovascular events, and mortality [4–6]. However, other research studies have shown that it is more cost-effective than staged-bilateral TKA, with better outcomes for the patient and lower costs [7]. Meanwhile, some other researchers observed that there was not much difference in complication rates between simultaneous and staged-bilateral TKA [5, 8, 9] and concluded that simultaneous bilateral TKA is a safe and effective procedure [10–12].

Many methods have been used during TKA to reduce blood transfusion and blood loss and shorten the operation time and time under anesthesia. Tranexamic acid (TXA) can be effective and safe for reducing blood transfusion and blood loss in simultaneous bilateral TKA without increasing thromboembolic complications in TKA [13, 14]. Many researchers have shown that TXA can reduce drainage volume [15, 16]. However, the optimal dosage and timing of TXA in simultaneous bilateral TKA are undetermined, with some research showing that there is significant benefit to using TXA in simultaneous bilateral TKA as a single injection [17–19] and other research indicating that multiple boluses of TXA after primary TKA without a tourniquet can reduce pain and knee swelling and lead to better knee function [20].

In addition to TXA, multimodal blood management protocols including preoperative hemoglobin evaluation, a high-protein diet, tourniquet release after skin closure, preoperative oral iron treatment, and femoral canal obturation can effectively reduce blood loss [21] and pain and knee joint swelling [22]. Tourniquets were used to reduce perioperative blood loss in TKA with many other advantages, such as clean and dry visualization of the surgical field [22–25]. However, tourniquets commonly used in TKA may contribute to pain [26] and worse knee function [26, 27].

In our study, TXA (10 mg/kg) was given intravenously to reduce perioperative blood loss [20], and the tourniquet was released after wound closure to reduce the duration of the TKA procedure and to avoid the possible risks of extended anesthesia in simultaneous bilateral TKA as previously reported [23].

In this research study, we found that the patients had different satisfaction levels with the first and second knees after simultaneous bilateral TKA, which is related to the order of TKA and not related to pre-operation symptomatic severity of disease.

To explore the reasons for this phenomenon, we retrospectively reviewed 46 patients who underwent simultaneous bilateral TKA in our department.

## Methods

We reviewed 46 patients who underwent simultaneous bilateral TKA between March 2013 and March 2015. Of these 46 patients, 38 were female and 8 were male. The mean age was 66 years old, ranging from 56 to 74 years old, and the mean body mass index was 27.2 kg/m^2^, ranging from 19.5 to 30.76 kg/m^2^.

The inclusion criteria were patients who were preoperatively diagnosed with severe bilateral knee osteoarthritis, and simultaneous bilateral TKA was used. The deformity of knees could not be more than ±10° with no need for soft tissue release during surgery, which is caused by an imbalance in the soft tissue. The patella could be resurfaced in both knees or not resurfaced in either knee.

The exclusion criteria were patients who underwent simultaneous bilateral TKA with a diagnosis other than osteoarthritis (including rheumatoid arthritis, pigmented villonodular synovitis, and others); those who could not understand and complete the pain visual analog scale (VAS) test; and patients who had severe psychiatric conditions, uncorrected visual acuity or hearing impairments, language difficulties, significantly different preoperative knee function or pain, or postoperative complications such as fracture around knees, lower extremity vascular diseases, and venous thromboembolism that could potentially affect postoperative outcome.

### Surgical technique

**Fig. 1:**
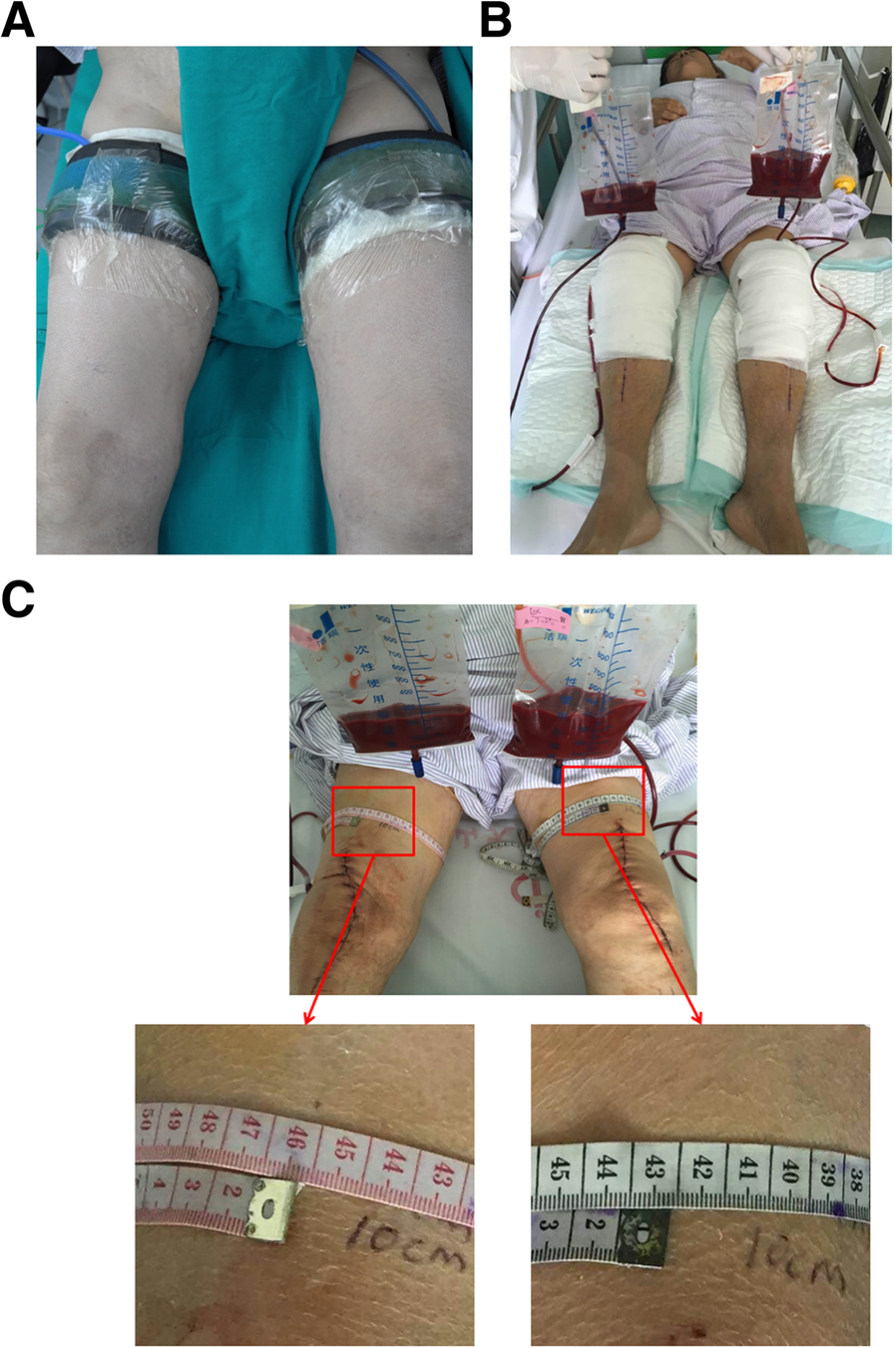
Surgical technique

All operations were performed in a sequential fashion by a single surgeon. Perioperative intravenous antibiotics were given 30 min prior to incision and were continued through postoperative day 2. The patient remained under epidural anesthesia throughout the entire surgery, the same set of instruments was used for both knees, and the right knee was operated upon first, as was the surgeon’s habit. A posterior-stabilized prosthesis (DePuy Synthes, P.F.C. Sigma (Warsaw, IN, USA)) was implanted in all patients. After elevation of the leg and inflation of the tourniquet, the knee was exposed using a midline skin incision with the traditional medial parapatellar approach. The inflation of the tourniquet was approximately 280 mm/Hg (Fig. 1a). The patella was resurfaced based on the patellofemoral orbit. After the first-side TKA was completed, the tourniquet was released, and TXA (10 mg/kg dose) was used to reduce blood loss. Then, the same operation procedure was used for the left knee. The wound was closed in layers over a dosed-suction drain and was wrapped with a sterile pad until the second knee procedure was completed. The knees of the patients were mobilized after the drains were pulled out within 24 h postoperatively, and the drainage volume and swelling were recorded (Fig. 1b and c). Then, ambulation and range of motion tests were initiated.

### Clinical evaluation

For clinical evaluation, the following objective and subjective parameters were collected:

Preoperative parameters

The American Knee Society knee score (KSS)

- Range of motion
- Pain VAS

Postoperative parameters

1. Drainage volume.
2. Degree of swelling in both knees: We measured the degree of swelling in both knees using the circumference 10 cm from the upper edge of the patella.
3. Satisfaction VAS: The scale consisted of a 100-mm-long horizontal line ranging from completely satisfied to totally unsatisfied. The satisfaction VAS score ranged from 0 (worst, totally unsatisfied) to 100 (best, completely satisfied) points [28].
4. Pain visual analog scale (VAS): The scale consisted of a 100-mm-long horizontal line ranging from no pain to intolerable pain. The pain VAS score ranged from 0 (best, no pain) to 100 (worst, intolerable pain) points [28].
5. Patient satisfaction: The parameters were collected preoperatively and at postoperative days 1, 3, and 7.

### Statistical analysis

Continuous variables were analyzed using Student’s *t* test. The *χ* ^2^ test and Fisher’s exact test were used to determine the statistical significance of differences in the categorical variables. The correlations among continuous variables were evaluated using Pearson correlation coefficients. Statistical analyses were performed using SPSS for Windows (version 21.0; SPSS). *p* < 0.05 was considered statistically significant.

## Results

### Preoperative parameters

In the 46 patients, none of the preoperative parameters were statistically significantly different (*p* > 0.05, Table 1). The data are presented as the means ± SD.

**Table 1:** Preoperative parameters

Variable	First knee	Second knee	*p* value
KSS	111.17 ± 5.01	111.17 ± 5.01	0.31
ROM (°)	107 ± 7.32	107.22 ± 8.3	0.814
Pain VAS	57.59 ± 8.42	58.86 ± 8.62	0.296

Data are presented as the mean ± SD

### Postoperative parameters

- In comparison to the second knee, the first knee had lower mean drainage volume, but the swelling of the first knee, measured as the circumference 10 cm from the upper edge of the patella, was higher on postoperative days 1, 3, and 7 (*p* < 0.05, Table 2).

**Table 2:** Objective measures

Variable	First knee	Second knee	*p* value
Tourniquet time (min)	85.3 ± 11.3	84.4 ± 10.2	0.352
Drainage volume (mL)	136.82 ± 30.76	433.18 ± 59.55	<0.001
Circumference 10 cm from the upper edge of the patella (cm)			
Preoperative	45.47 ± 2.26	45.36 ± 2.30	0.247
Postoperative day 1	49.83 ± 2.33	47.60 ± 2.50	<0.05
Postoperative day 3	48.15 ± 2.35	45.42 ± 2.30	<0.05
Postoperative day 7	46.25 ± 2.37	45.76 ± 2.34	<0.05

Data are presented as the mean ± SD
- Comparison of the results of the satisfaction and pain VAS between the first and second knee on postoperative days 1, 3, and 7 are illustrated in Table 3. The first knee had lower satisfaction VAS scores but higher pain VAS scores.

**Table 3:** Subjective measures

Variable	First knee	Second knee	*p* value
Satisfaction VAS			
Postoperative day 1	74.41 ± 5.50	81.91 ± 3.58	<0.05
Postoperative day 3	78.68 ± 4.05	85.09 ± 3.66	<0.05
Postoperative day 7	83.32 ± 3.63	89.64 ± 4.39	<0.05
Pain VAS			
Postoperative day 1	56.59 ± 17.55	44.09 ± 19.11	<0.05
Postoperative day 3	42.00 ± 15.32	33.64 ± 16.20	<0.05
Postoperative day 7	34.41 ± 11.25	24.82 ± 11.91	<0.05

Data are presented as the mean ± SD
- The patient satisfaction scores indicated 2 (4.4%) of 46 patients scored the first knee higher than the second knee and 34 (73.9%) of 46 patients scored the second knee higher than the first knee. Ten (21.7%) of 46 patients scored their satisfaction as the same in both knees.

## Discussion

Osteoarthritis of the knee often includes bilateral involvement and requires TKA, but choosing staged or simultaneous bilateral TKA is still controversial. Studies have shown that simultaneous bilateral TKA is an effective treatment for patients with osteoarthritis, and it reduces hospital expense and recovery time [4–7, 29]. Whether there are satisfaction level differences with the first and second knees in the early stage after simultaneous bilateral TKA is still unknown. In our research, one of the important findings is that there is better satisfaction with the second knee in the early stage after simultaneous bilateral TKA. We found that the second knee had higher satisfaction VAS and lower pain VAS, drainage volume, and swelling compared with the first knee.

Bullens et al. [28] reported that the satisfaction VAS scores provided additional information about subjective outcomes after TKA and were lower in the first knee than the second knee. Satisfaction VAS scores mirrored patient satisfaction and results from the objective parameters.

In fact, surgeons have different methods for using TXA and tourniquets during simultaneous bilateral TKA, which may affect patient satisfaction with both knees in the postoperative period. Many methods have been used in simultaneous bilateral TKA to reduce blood transfusion and blood loss and shorten operation time and time under anesthesia, such as the use of TXA and tourniquets. However, the different dosages and timing of TXA in TKA can affect pain, knee swelling, and knee function, and tourniquets may also contribute to pain [26] and worse knee function [26, 27]. Therefore, whether TXA and tourniquets in simultaneous bilateral TKA affected pain, knee swelling, and knee function are still unknown.

Furthermore, the optimal dosage and timing of TXA in TKA remain undetermined. TXA (10 mg/kg) was given intravenously to reduce perioperative blood loss [20]. A fixed dose of TXA for patients undergoing simultaneous bilateral TKA was also effective and safe in reducing total blood loss and allogeneic blood transfusion needs without any additional thromboembolic risk [15].

However, there has been no report of drainage volume differences in simultaneous bilateral TKA. Our records showed that the average drainage volume in the first knee was less than that in the second one, which may be due to the timing of TXA between the completion of the prosthesis installation and closing the incision for the first-side TKA. Additionally, there is less published research available for drainage volume, which was statistically significantly different in both knees after simultaneous bilateral TKA.

Multiple boluses of TXA (3 and 6 h after surgery) without a tourniquet can reduce pain and knee swelling and lead to better knee function compared with single boluses before skin incision [20]. However, the TXA used in our study was provided between the completion of the prosthesis installation and closing the incision. Therefore, it would affect the first knee by providing TXA when the right tourniquet was released to accompany the blood congestion in the first knee. We know that the half-period of TXA is 2 h and the peak time is 3 h after injection. Therefore, the first knee had more time with TXA in our procedure, especially after the left limb blood flow was removed. Therefore, the timing of TXA administration may be one of the reasons for the decreased mean drainage volume in the first knee compared to the second knee, resulting in the different amounts of swelling in both knees.

Moreover, a previous study reported that the control of swelling could reduce pain and improve the rapid rehabilitation of the knee after surgery [30]. Noble et al. [31] also suggested that improvements in the results of TKA would prevent the knees from swelling. Therefore, in our study, we found that the swelling of the first knee was higher than that of the second after surgery, which may have led to higher pain VAS scores and the lower satisfaction in the first knee.

A tourniquet release after wound closure was used to reduce the duration of the TKA procedure and to avoid the possible risks of extended anesthesia in simultaneous bilateral TKA as previously reported [23]. A tourniquet was used to reduce perioperative blood loss in TKA with many other advantages, such as clean and dry visualization of the surgical field [22–25]. However, the current evidence is not enough to indicate that tourniquet release before wound closure is superior to its release after wound closure in cemented TKA [24]. Moreover, tourniquets commonly used in TKA may contribute to pain [26] and worse knee function [26, 27]. A meta-analysis showed that TKA with a tourniquet might hinder patients’ early postoperative rehabilitation exercises [25]. Furthermore, limb swelling and knee joint pain may affect the use time of a tourniquet in TKA [32]. Tai et al. [33] reported that there were no significant differences in swelling defined as the circumference of the limb between the tourniquet and no-tourniquet areas, but the time spent using the tourniquet was 52.5 ± 10 min, which may have minimally affected the knee. In our surgery, the time spent using the tourniquet was 85.3 ± 11.3 min in the first-side TKA and 84.4 ± 10.2 min in the second side. However, we used an unsterile tourniquet, which was released after the first knee surgery was performed but not removed from the limb until the end of the bilateral TKA. This action might have affected the blood deposit in the vein and resulted in swelling, especially due to the fragility of the imbalanced condition after the surgery.

Currently, there is a type of sterile tourniquet that can be released when the surgery is completed, which may help to reduce the satisfaction level difference of patients in the early stage of simultaneous bilateral total knee arthroplasty.

There are some limitations to this research study. First, the correlation among patient satisfaction, leg swelling, and the patients’ pain was not examined. Second, we did not use ultrasonography to confirm whether the swelling was due to the DVT. However, the patients had no signs or symptoms of thromboembolism, so it was not a routine check. Furthermore, the long-term clinical outcomes of both knees will be followed in the future.

## Conclusions

This research study found better patient satisfaction levels with the second knee in the early stage after simultaneous bilateral TKA, which may offer some considerations for surgeons choosing simultaneous bilateral total knee arthroplasty in patients with osteoarthritis in both knees.

## Abbreviations

TKA: Total knee arthroplasty
TXA: Tranexamic acid
KSS: Knee Society knee score
VAS: Visual analog scale
